# Neurofilament Light Chain Protein Is a Predictive Biomarker for Stroke After Surgical Repair for Acute Type A Aortic Dissection

**DOI:** 10.3389/fcvm.2021.754801

**Published:** 2021-11-11

**Authors:** Kai Zhang, Zhu Wang, Kai Zhu, Songbo Dong, Xudong Pan, Lizhong Sun, Qing Li

**Affiliations:** ^1^Department of Cardiothoracic Surgery, The Affiliated Hospital of Xuzhou Medical University, Xuzhou, China; ^2^Jiangsu Provincial Institute of Health Emergency, Xuzhou Medical University, Xuzhou, China; ^3^Department of Cardiovascular Surgery, Beijing Anzhen Hospital, Capital Medical University, Beijing, China

**Keywords:** type A aortic dissection, aortic operation, stroke, biomarker (BM), neurofilaments (NFs)

## Abstract

**Background:** Although great progress has been made in surgery and perioperative care, stroke is still a fatal complication of acute type A aortic dissection (ATAAD). Serum biomarkers may help assess brain damage and predict patient's prognosis.

**Methods:** From March, 2019 to January, 2020, a total of 88 patients underwent surgical treatment at the Department of Cardiovascular Surgery of Beijing Anzhen Hospital, China, and were enrolled in this study. Patients were divided into two groups according to whether they had suffered a stroke after the operation. Blood samples were collected at 8 time points within 3 days after surgery to determine the level of S100β, neuron-specific enolase (NSE) and neurofilament light chain protein (NFL). Receiver operating characteristic curves (ROC) were established to explore the biomarker predictive value in stroke. The area under the curve (AUC) was used to quantify the ROC curve.

**Results:** The patient average age was 48.1 ± 11.0 years old and 70 (79.6%) patients were male. Fifteen (17.0%) patients suffered stroke after surgery. The NFL levels of patients in the stroke group at 12 and 24 h after surgery were significantly higher than those in the non-stroke group (all *P* < 0.001). However, the NSE and S100β levels did not differ significantly at any time point between the two groups. The predictive value of NFL was the highest at 12 and 24 h after surgery, and the AUC was 0.834 (95% CI, 0.723–0.951, *P* < 0.001) and 0.748 (95% CI, 0.603–0.894, *P* = 0.004), respectively. Its sensitivity and specificity at 12 h were 86.7 and 71.6%, respectively. The NFL cutoff value for the diagnosis of stroke at 12 h after surgery was 16.042 ng/ml.

**Conclusions:** This study suggests that NFL is an early and sensitive serum marker for predicting post-operative neurological prognosis of ATAAD patients. Further studies, including large-scale prospective clinical trials, are necessary to test whether the NFL can be used as a biomarker for clinical decision-making.

## Introduction

Acute type A aortic dissection (ATAAD) is a challenging condition that requires complex and life-saving cardiovascular surgery. Although the success rate of surgery has improved in recent years (1), post-operative neurological complications unfortunately still occur. Therefore, there is a clinical need to unravel specific biomarkers that can help identify irreversible nerve damage in the early post-operative period, so treatment strategies can be actioned in a timely manner.

Previously, serum neuron-specific enolase (NSE) and S100β protein were considered promising candidate marker proteins for predicting the neurological status of patients after cardiac surgery (2–4), however, studies on these two markers yielded conflicting results (5, 6). More importantly, these two markers are not only expressed in neurons but also in other; NSE is expressed in red blood cells and platelets, and S100β is expressed in fat and other tissues or organs (7–9). This lack of specificity makes it difficult to identify changes in serum levels that are specifically associated with brain damage.

Neurofilament light chain protein (NFL) is the main component of neurofilament core and the main intermediate filament protein of neurons and axons (10, 11). Rosén et al. (12) suggested that the NFL level in the cerebrospinal fluid has a high degree of sensitivity and specificity in predicting brain damage in patients after cardiac arrest. However, due to the various complications and contraindications of lumbar puncture, its usage has been limited. On this basis, Moseby-Knappe et al. (13) have investigated the biomarker value of NFL in blood samples and found that, similarly to the cerebrospinal fluid, the increase in NFL levels in blood samples is also associated with the poor neurological results after cardiac arrest. However, to date there are no data on serum biomarker levels in stroke patients after ATAAD. In order to find a reliable and specific biomarker that can help predict post-operative neurological results in patients who suffered stroke, we conducted a prospective observational cohort study and investigated changes in serum S100β, NSE, and NFL levels after surgery.

## Methods

### Study Design

This study was approved by the Ethics Committee of Beijing Anzhen Hospital, Capital Medical University, China, and complies with the requirements of the Good Clinical Practice international guidelines. All procedures are within the normal routine intensive care routine with no additional risk to the patients, therefore the institutional review board abandoned the need to obtain patient's informed consent. The research protocol complies with the ethical guidelines of the 1975 Declaration of Helsinki. The data for this observational cohort study was collected in a prospective manner. Patients who met the inclusion criteria (see below) were admitted to the theater, blood samples were collected to determine the level of biomarkers at 8 time points: 5 min after induction of anesthesia (T0), at the beginning of selective cerebral perfusion (T1), at the time weaned from cardiopulmonary bypass (CPB) (T2), 6 h after operation (T3), 12 h after operation (T4), 24 h after operation (T5), 48 h after operation (T6), and 72 h after operation (T7). Blood samples were collected independently by two clinicians, neither of who was responsible for the treatment of the patients. In the process of treating patients, no clinicians had access to relevant information about S100β, NSE, and NFL levels. Patients were divided into groups according to whether they had suffered a stroke after the operation. Post-operative stroke was defined as the development of new global or focal neurological deficits within 30 days post-surgery, as assessed by cranial CT scan. Based on previous studies (14, 15), malperfusion syndrome is defined as compromised blood flow in 1 or more organs resulting in ischemia and organ dysfunction due to the dissection-related obstruction of the aorta and its branch vessels.

### Patients

Patients with ATAAD who underwent surgery at the Department of Cardiovascular Surgery of Beijing Anzhen Hospital, China, were prospectively included in the study. Inclusion criteria were: age > 18 years old; underwent total arch replacement (TAR), and stented elephant trunk (SET) implantation. Exclusion criteria were: continuous coma before surgery, and lack of at least 4 of 8 blood samples.

### Surgical Techniques

All operations were performed through a median sternum incision. Briefly, operations used the right axillary artery cannulation for cardiopulmonary bypass and selective cerebral perfusion (5–10 ml/kg·min) under hypothermic circulatory arrest (HCA). First, proximal aortic root operations, including ascending aorta replacement or Bentall procedure, were completed. If surgery was combined with concomitant operations, it was completed during the cooling period. As stated in our previous studies (16, 17), we used TAR + SET implantation to treat aortic arch, which included implanting SET in the descending aorta (Cronus, MicroPort, China), and then using four branched graft (Vascutek, Terumo, Japan) to complete TAR. After the descending aorta anastomosis was completed, the distal reperfusion was started. First, the left carotid artery was reconstructed to achieve bilateral perfusion, then the ascending aorta was reconstructed to prevent coronary ischemia, and finally the subclavian artery and the innominate artery were reconstructed.

### Biomarker Measurements

All tests complied with the standard biosecurity and institutional safety procedures. At each of the blood collection time points, 2 ml of blood from the central venous tube was drawn and discarded, then 5 ml of blood was drawn and collected into a tube containing ethylenediamine tetra acetic acid, and kept refrigerated at 4°C. Then, the sample was centrifuged at 3,000 rpm for 10 min and the serum was collected and stored at −80°C. The levels of S100, NSE and NFL were analyzed by a commercial enzyme-linked immunosorbent assay (R&D Systems, Minneapolis, MN). Samples were run on TECAN Infinite F50 (TECAN group, Männedorf, Switzerland). The detection limit was specified by the manufacturer. All tests were carried out by laboratory technicians blind to the clinical data. All tests are performed in duplicate, and the mean was calculated and used for analysis.

### Statistical Analysis

Categorical variables were expressed as numbers (percentage). Continuous variables conforming to a normal distribution were expressed as mean ± SD, or expressed as the median (interquartile range). Comparisons were performed using the Student's *t*-test, Mann-Whitney *U*-test, Chi-square-test, or Fisher's exact-test. The generalized additive mixed model was used to compare the NFL levels of the two groups at each time point. Receiver operating characteristic curves (ROC) were generated to provide data on the predictive ability of biomarkers to detect stroke. The area under the curve (AUC) was used to quantify the ROC curve. Youden's index (*J* = Sensitivity + Specificity-1) was used to determine the most appropriate cut-off value. All tests were two-sided, with *P* < 0.05 as the significance threshold. GraphPad Prism 8 for Windows (GraphPad software, La Jolla, CA, USA) was used for graphing. R and Empower Stats (http://www.empowerstats.com, X&Y Solutions, Inc., MA, USA) were used for all statistical analyses.

## Results

### Pre-operative and Intraoperative Data of Patients

From 14 March, 2019 to 19 January, 2020, a total of 108 patients underwent surgical treatment in the Department of Cardiovascular Surgery of Beijing Anzhen Hospital, Beijing, China. Ninety-nine met the eligibility criteria, and nine were excluded. Eleven patients were excluded at a later stage due to poor sample quality or hemolysis, missing samples, or sample transportation problems. In total, we collected data on 88 consecutive cases, of which 15 (17.0%) suffered post-operative stroke. Three patients suffered a stroke on the second day after surgery, four patients on the third day after surgery, and two patients on the fourth day after surgery. The remaining six patients suffered a stroke on the 5th, 6th, 7th, 9th, 10th, and 11th post-operative day.

As shown in Table 1, the average age of the patients was 48 years old, the average BMI was 27.3, 70 cases (79.6%) were male, and emergency operations accounted for 94.3% of all operations. The most common comorbidity was hypertension in 74 cases (84.1%). Smoking history was present in 45 cases (51.1%). The average CPB time was 198.9 ± 34.8 min, the aortic cross-clamp time was 113.2 ± 25.6 min, and the HCA time was 24.2 ± 9.8 min. Among root treatment methods, Bentall procedure accounted for 25 cases (28.4%). There were 11 cases of concomitant operations, including three cases of CABG (3.4%) and eight cases of bypass (9.1%).

**Table 1:** Pre-operative and intraoperative characteristics.

**Variables**	**Total (*n* = 88)**	**Non-stroke (*n* = 73)**	**Stroke (*n* = 15)**	***P-*value**
Age	48.1 ± 11.0	47.8 ± 11.5	50.0 ± 8.2	0.479
Gender				0.453
Male	70 (79.5)	57 (78.1)	13 (86.7)	
Female	18 (20.5)	16 (21.9)	2 (13.3)	0.228
BMI (kg/m^2^)	27.3 ± 4.5	27.0 ± 4.6	28.6 ± 4.2	
Diabetes mellitus	1 (1.1)	0	1 (6.7)	0.170
Hypertension	74 (84.1)	61 (83.6)	13 (86.7)	0.765
Coronary artery disease	3 (3.4)	3 (4.1)	0	0.424
Prior cerebrovascular accident	5 (5.7)	3 (4.1)	2 (13.3)	0.160
Chronic renal insufficiency	2 (2.3)	2 (2.7)	0	0.517
Prior cardiovascular intervention	3 (3.4)	3 (4.1)	0	0.424
Smoking history	45 (51.1)	35 (47.9)	10 (66.7)	0.186
Drinking history	33 (37.5)	28 (38.4)	5 (33.3)	0.714
Malperfusion syndrome	26 (29.5)	18 (24.7)	8 (53.3)	0.027
Ejection fraction	63.3 ± 4.8	63.5 ± 5.0	62.0 ± 4.0	0.261
Aortic regurgitation (moderate or severe)	23 (26.2)	16 (21.9)	7 (46.7)	0.047
Emergency	83 (94.3)	68 (93.2)	15 (100.0)	0.297
Operation time (h)	7.4 ± 1.3	7.3 ± 1.4	7.8 ± 0.6	0.178
Lowest nasal temperature (°C)	25.1 ± 1.8	25.1 ± 1.9	24.8 ± 1.2	0.554
Lowest bladder temperature (°C)	26.7 ± 1.8	26.8 ± 1.9	26.2 ± 1.2	0.237
CPB time (min)	198.9 ± 34.8	195.2 ± 35.5	216.7 ± 25.2	0.028
Cross-clamp time (min)	113.2 ± 25.6	109.7 ± 24.3	129.9 ± 25.8	0.005
Lower body circulatory arrest time (min)	24.2 ± 9.8	24.2 ± 9.4	24.2 ± 12.0	0.994
Selective cerebral perfusion time (min)	34.2 ± 10.9	33.6 ± 11.0	37.5 ± 10.5	0.201
Aortic root repair				0.642
Ascending aorta replacement	63 (71.6)	53 (72.6)	10 (66.7)	
Bentall procedure	25 (28.4)	20 (27.4)	5 (33.3)	
Concomitant operation				
Extra-anatomic bypass	8 (9.1)	7 (9.6)	1 (6.7)	0.720
CABG	3 (3.4)	3 (4.1)	0	0.424
Intraoperative PLT transfusion	4 (4.5)	3 (4.1)	1 (6.7)	0.665
Intraoperative RBC transfusion	18 (20.5)	16 (21.9)	2 (13.3)	0.453
Intraoperative FFP transfusion	21 (23.9)	18 (24.7)	3 (20.0)	0.700

*Data represent n (%), mean ± SD, or median (interquartile range)*.

Compared with the non-stroke group, patients who had developed stroke after surgery had a higher pre-operative rate of moderate/severe aortic regurgitation (7/15, 46.7% vs. 16/73, 21.9%; *P* < 0.05), and malperfusion syndrome (8/15, 53.3% vs. 28/73, 38.4%; *P* < 0.05). In terms of intraoperative data, patients in the stroke group experienced significantly longer CPB time and aortic cross-clamp time (216.7 ± 25.2 vs. 195.2 ± 35.5 min, 129.9 ± 25.8 vs. 109.7 ± 24.3 min; all *P* < 0.05). Other analysis revealed no statistical difference between the two groups.

### Post-operative Mortality and Morbidity

Table 2 shows the rate of post-operative complications of these patients. The overall mortality rate of the patients was 12.5% (11/88). Five of the patients who died had a stroke after surgery, accounting for 33.3% of the patients in the Stroke group (*P* < 0.05). There were 14 cases of continuous renal replacement therapy (15.9%), two cases of paraplegia (2.3%), and five cases (5.7%) of re-examination for bleeding. Although the length of hospital stay was not statistically different between groups, the ICU time of the stroke group was longer than that of the non-stroke group (*P* < 0.001).

**Table 2:** Patient mortality and morbidity.

**Variables**	**Total (*n* = 88)**	**Non-stroke (*n* = 73)**	**Stroke (*n* = 15)**	***P-*value**
ICU stay time (h)	60.6 (22.7–114.2)	40.5 (18.7–90.0)	180.5 (84.0–278.2)	<0.001
Hospital stay time (d)	13.0 (10.0–16.2)	10.0 (13.0–16.0)	14.0 (10.0–18.5)	0.954
Tracheostomy	7 (8.0)	4 (5.5)	3 (20.0)	0.058
Re-exploration for bleeding	5 (5.7)	4 (5.5)	1 (6.7)	0.856
CRRT	14 (15.9)	11 (15.1)	3 (20.0)	0.634
Paraplegia	2 (2.3)	2 (2.7)	0	0.517
Gastrointestinal bleeding	5 (5.7)	4 (5.5)	1 (6.7)	0.856
Mortality	11 (12.5)	6 (8.2)	5 (33.3)	0.007

*Data represent n (%), mean ± SD, or median (interquartile range)*.

### Serum Biomarker Levels

**Figure 1:**
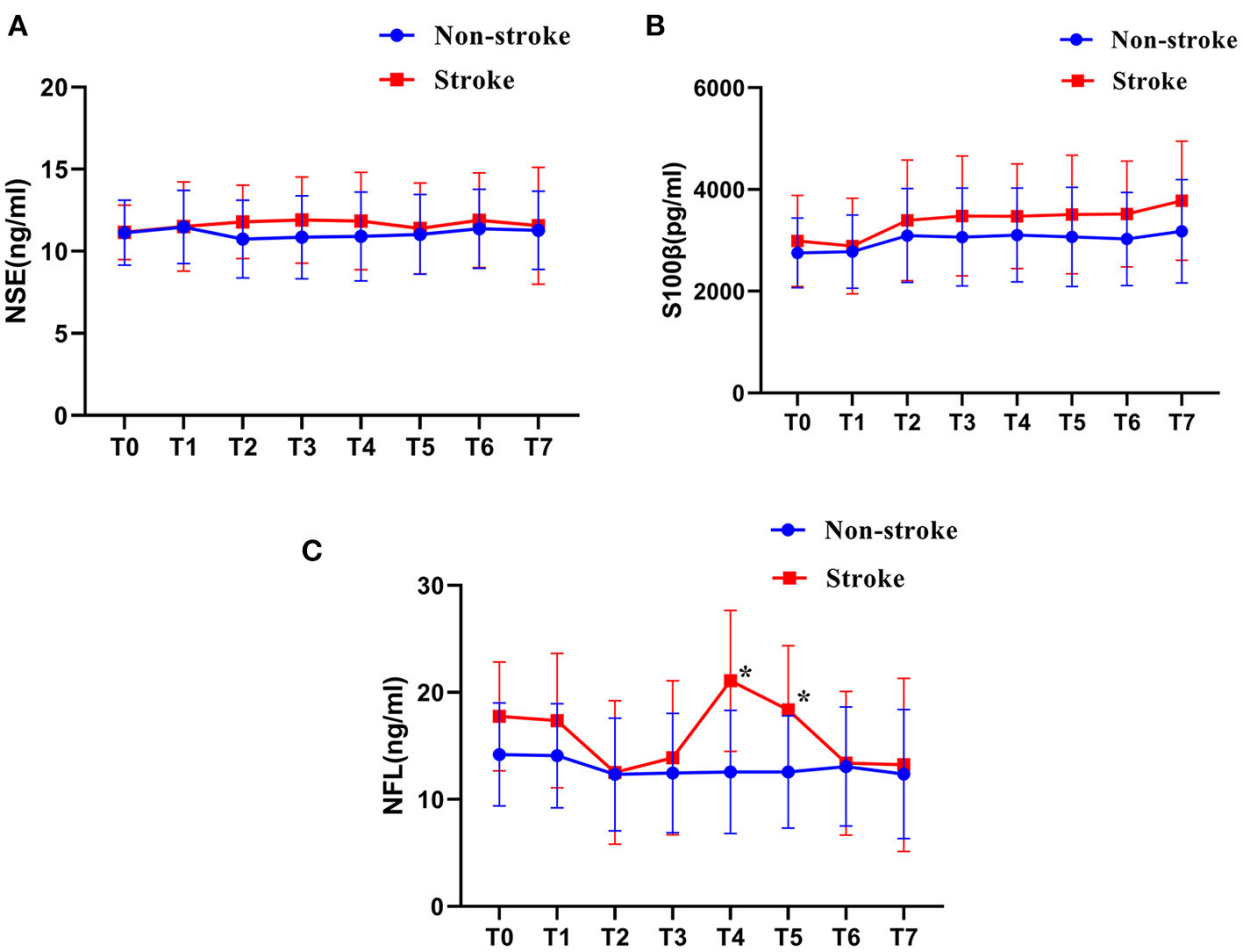
**(A–C)** Biomarker comparison across groups and time points ^*^P < 0.001.

A total of 535 samples were collected at 5 min after induction of anesthesia (T0), at the beginning of selective cerebral perfusion (T1), at the time of weaning from CPB (T2), 6 h after operation (T3), 12 h after operation (T4), 24 h after operation (T5), 48 h after operation (T6), and 72 h after operation (T7). As shown in Supplementary Table S1 and in Figure 1C, the levels of NFL in patients in the stroke group were significantly higher than those in the non-stroke group at 12 and 24 h after surgery (all *P* < 0.001). However, the NSE levels did not differ significantly at any time point between the two groups (Figure 1A). Although the average S100β level of stroke patients was higher in patients who had a stroke than in patients who did not have a stroke at all study time points, the difference was not significant (all *P* > 0.05; Figure 1B).

### Serum NFL Predictive Ability

**Figure 2:**
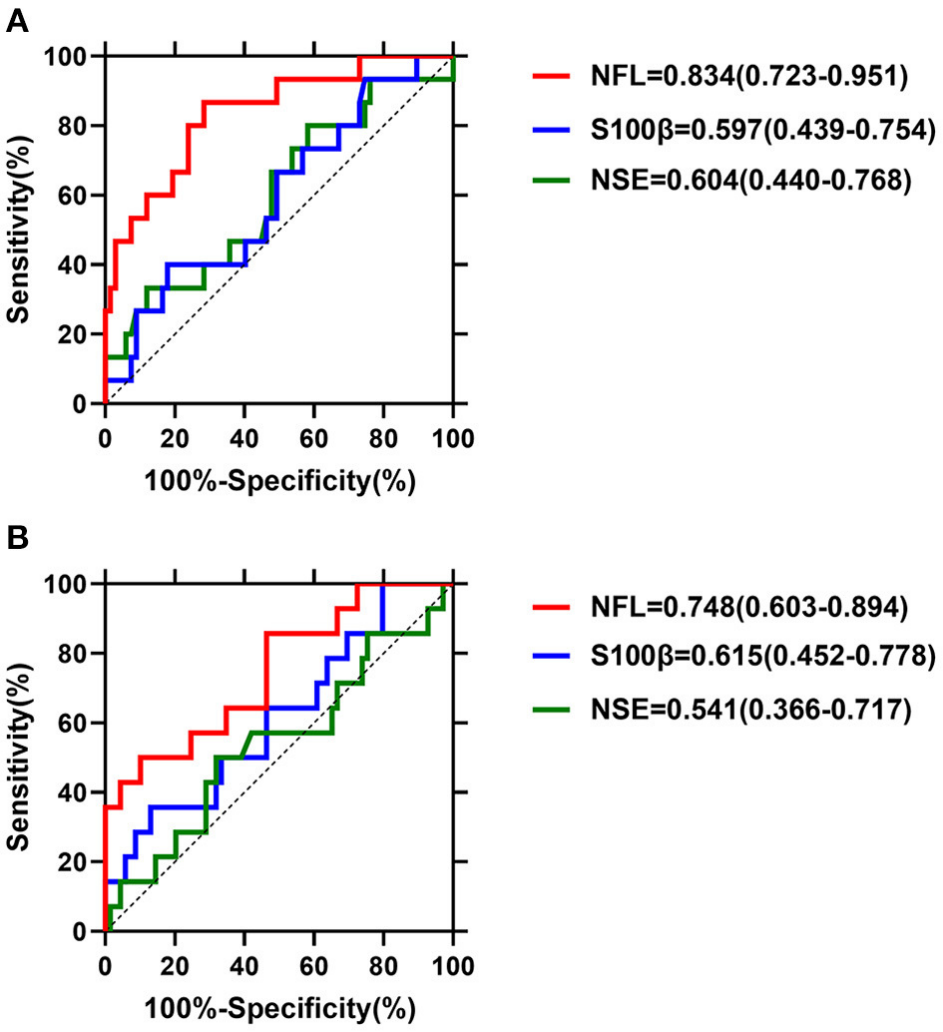
**(A,B)** Biomarker accuracy for predicting post-operative stroke.

We performed ROC analysis and AUC, and measured the biomarker cutoff value, sensitivity, specificity and predictive value at T4 and T5. As shown in Figures 2A,B, S100β and NSE did not demonstrate a good predictive ability. As shown in Table 3, the predictive value of NFL was the highest at 12 h (T4) and 24 h (T5) after surgery, with an AUC of 0.834 (95% CI, 0.723–0.951, *P* < 0.001) and 0.748 (95% CI, 0.603–0.894, *P* = 0.004), respectively. Its sensitivity and specificity at 12 h after surgery were 86.7 and 71.6%, respectively. The NFL cutoff value for the diagnosis of stroke at 12 h after surgery was 16.042 ng/ml. These analysis indicate the value of NFL holds as a predictor of stroke risk.

**Table 3:** Cut-off points, sensitivities, specificities, and AUC values of NFL levels for stroke prediction by ROC analysis.

**Variables**	**Cut-off value (ng/ml)**	**Specificity**	**Sensitivity**	**AUC**	**95% CI of AUC**	***P-*value**
NFL-T4	16.042	0.716	0.867	0.834	0.723–0.951	<0.001
NFL-T5	19.339	0.899	0.500	0.748	0.603–0.894	0.004

## Discussion

In summary, this prospective study evaluated the level changes and diagnostic accuracy of the biomarkers S100β, NSE, and NFL at 8 different time points in 3 days in ATAAD patients treated with TAR+FET in our cardiovascular surgery center. This study is one of the first and largest studies to evaluate the temporal profiles of these three biomarkers in ATAAD patients with and without stroke. The main findings of this study are: (1) After surgical repair of ATAAD, the serum NFL level of stroke patients is increased in comparison with patients with a good neurological prognosis, (2) Serum NFL level appears to hold more predictive value than the conventional biomarkers S100β and NSE, (3) Serum NFL level is a more reliable and sensitive predictor of stroke after surgical repair of ATAAD. NFL level at 12 h after surgical repair of ATAAD may become an important tool for detecting stroke, potentially complement existing methods neurological prognosis for assessment and help make earlier clinical predictions.

In the past, NFL was rarely used in the assessment of neurological prognosis in the field of cardiac surgery. Alifier et al. (18) found that patients who had cardiac surgery showed higher NFL levels than those underwent other operations, and patients who experienced CPB had even higher levels. In a study conducted by Saller et al. (19) patients who had undergone cardiac surgery were divided into three groups (off-pump coronary artery bypass without delirium group, CPB without delirium group and CPB with delirium group). The authors observed a sharp increase in NFL levels in patients suffering post-operative delirium after CPB, and speculated that surgery or trauma could cause systemic inflammation, inducing a neuroinflammatory response and microglia activation, eventually leading to neuronal damage (20).

Currently, little is known about the changes in serum NFL levels during the perioperative period of ATAAD, so we set out to investigate this question. In this study, the median NFL level of patients who had a stroke 12 h after ATAAD was almost double of that of patients who did not have a stroke (21.4 vs. 11.8 ng/ml), and it was still significantly higher than that of non-stroke patients at 24 h. However, it decreased rapidly at 48 h after ATAAD, a finding that appears inconsistent with the slow change of NFL reported in other studies (13, 18). For example, it may take weeks or months for NFL levels to return to normal after traumatic brain injury, as reported in a study in a population of professional boxers (21). Interestingly, although there was no statistical difference, patients in the stroke group had a higher baseline value (T0) than those in the non-stroke group. According to previous studies, diseases such as amyotrophic lateral sclerosis, multiple sclerosis, and HIV-associated dementia may significantly increase NFL serum levels and affect the predicted value of NFL (22–24). Although the patients enrolled in our study had no reported history of such medical conditions, these factors should be taken into consideration when evaluating patients. In addition, we suggest that there might be other explanations for this seemingly inconsistent finding. As a major cause of stroke, cerebral small vessel disease (CSVD) is one of the most frequent pathologic conditions neurologists can encounter. A recent study (25) reported a two-fold increase of serum NFL levels in CSVD subjects compared with healthy controls, an association was observed with both imaging and clinical features. Gattringer et al. (26) found that serum NFL levels have also been associated with the occurrence of small subcortical infarcts and with new-CSVD-related MRI lesions, suggesting NFL levels could act as a putative marker of active CSVD. Age constitutes a major risk factor for CSVD, and vascular aging is very frequently accompanied by chronic major vascular conditions for CSVD, including hypertension, diabetes, and smoking. All of these conditions and comorbidities are present in the patients included in this study and are more common in the stroke group. Since ATAAD patients often require emergency surgery and lack adequate pre-operative examination, CSVD might be related to the actual underlying conditions of these patients.

An ideal biomarker for brain injury should be both specific and sensitive. When the central nervous system (CNS) is injured, it can pass through the blood-brain barrier, leading to increased biomarker levels in the blood (27). S100β is widely expressed in various glial cells of the CNS in mammals, and previous studies have shown satisfactory sensitivity and specificity for serum S100β in the diagnosis of ischemic stroke after cardiac surgery (2). Similarly, as an enzyme of glycolysis, there are many clinical studies that support the use of NSE as a marker of brain injury after CPB (3, 4). However, in our study, when comparing NFL with NSE and S100β, NFL was the only marker that predicted stroke accurately at 12 h after surgery, with a better accuracy at 24 h than other biomarkers. The reason for this difference may be that NSE is susceptible to hemolysis (7). The application of S100β has also been questioned due to its expression in extracranial tissue sources such as fat cells and chondrocytes (8, 9).

Although surgical techniques, anesthesia and extracorporeal circulation management, post-operative monitoring and even artificial blood vessel materials have all greatly improved in the past 20 years, strokes after ATAAD are still relatively common, with an incidence of about 10–30% (28, 29). In this study, the incidence of post-operative stroke in the entire cohort was 15%. Of the 11 patients who died, five had post-operative stroke, which seriously affected their quality of life, endangered their lives, and brought great economic and spiritual burdens to their family. Prior literature has reported a variety of mechanisms involved in brain injury after ATAAD including the formation of embolus (atherosclerotic plaque, air, or blood clots) originating from the aorta or CPB circuit, brain hypoperfusion during CPB, and hypoperfusion related to systemic inflammation (30). However, it is difficult to evaluate the patient's neurological condition during surgery and during the early post-operative period for a number of reasons: due to the effects of post-operative sedation and mechanical ventilation, coupled with unstable vital signs, moving the patient may not be appropriate, and traditional “gold standard” examinations such as CT and MRI are restricted; due to the influence of drugs, EEG data may not be accurate and near infrared spectroscopy is also of limited value in continuous monitoring of brain tissue oxygen saturation (31). Peripheral blood biomarkers that reflect brain damage could provide objective quantitative measures to help guide clinical practice and management, for example by improving the assessment of damage and contributing to a timely and accurate diagnosis.

This study also has limitations. First, the sample size was relatively small, and therefore larger prospective studies with longer follow-up are needed. Second, our study was a prospective observational study, and therefore randomized controlled trials are necessary to validate the results. Before the serum NFL level can be used for clinical decision-making in ATAAD patients, it is important that the cut-off values are validated and a reliable laboratory reference range established.

## Conclusions

This study suggests for the first time the value of serum NFL as an early and sensitive serum marker for predicting post-operative stroke of ATAAD patients, particularly 12 h after surgery. Our findings should be validated by large-scale prospective clinical trials to test whether serum NFL levels have a value in clinical decision-making.

## Data Availability Statement

The original contributions presented in the study are included in the article/Supplementary Material, further inquiries can be directed to the corresponding author.

## Ethics Statement

The studies involving human participants were reviewed and approved by the Ethics Committee of Beijing Anzhen Hospital, Capital Medical University. Written informed consent for participation was not required for this study in accordance with the national legislation and the institutional requirements.

## Author Contributions

KZha, XP, and QL: design the research. KZha, SD, KZhu, and LS: analyze the data. KZha, ZW, and QL: write the article. All authors contributed to the article and approved the submitted version.

## Funding

This study was supported in part by Beijing Municipal Natural Science Foundation (No. 7202038).

## Conflict of Interest

The authors declare that the research was conducted in the absence of any commercial or financial relationships that could be construed as a potential conflict of interest.

## Publisher's Note

All claims expressed in this article are solely those of the authors and do not necessarily represent those of their affiliated organizations, or those of the publisher, the editors and the reviewers. Any product that may be evaluated in this article, or claim that may be made by its manufacturer, is not guaranteed or endorsed by the publisher.
