# Supplementary material for: Neurofilament Light Chain Protein Is a Predictive Biomarker for Stroke After Surgical Repair for Acute Type A Aortic Dissection
Source: Front Cardiovasc Med. 2021 Nov 11;8:754801. doi: 10.3389/fcvm.2021.754801 (PMC8631920; doi:10.3389/fcvm.2021.754801)
Supplement: Supplementary file 1 [file Table_1.docx]

# Complimentary information-TABLE S1. Biomarker levels at different time points.

| Variables | Non-stroke (n=73) | Stroke (n=15) | P value |
| --- | --- | --- | --- |
| T0 | n=42 | n=6 |  |
| S100β, pg/ml | 2628.5 (2281.2-3170.2) | 2906.0 (2419.8-3167.2) | 0.452 |
| NSE, ng/ml | 11.3 (10.0-12.3) | 10.8 (10.6-11.9) | 0.981 |
| NFL, ng/ml | 14.4 (10.8-17.8) | 19.2 (17.7-20.5) | 0.100 |
| T1 | n=34 | n=5 |  |
| S100β, pg/ml | 2642.5 (2395.2-3256.0) | 2708.0 (2231.0-2916.0) | 0.762 |
| NSE, ng/ml | 11.4 (9.6-13.1) | 10.2 (9.6-12.8) | 0.980 |
| NFL, ng/ml | 13.9 (11.1-17.6) | 20.9 (17.2-21.0) | 0.183 |
| T2 | n=59 | n=12 |  |
| S100β, pg/ml | 2878.0 (2363.0-3522.5) | 3068.0 (2283.8-4748.2) | 0.336 |
| NSE, ng/ml | 11.2 (9.1-12.4) | 11.5 (9.7-13.9) | 0.165 |
| NFL, ng/ml | 12.6 (7.6-16.0) | 12.6 (7.6-18.4) | 0.912 |
| T3 | n=66 | n=12 |  |
| S100β, pg/ml | 2830.0 (2338.2-3530.0) | 3100.0 (2574.0-4588.2) | 0.189 |
| NSE, ng/ml | 10.8 (8.9-12.9) | 11.1 (10.2-14.0) | 0.191 |
| NFL, ng/ml | 12.9 (7.1-16.8) | 16.1 (7.9-19.6) | 0.440 |
| T4 | n=67 | n=15 |  |
| S100β, pg/ml | 2972.0 (2342.5-3586.0) | 3029.0 (2717.5-4460.5) | 0.175 |
| NSE, ng/ml | 10.8 (8.9-13.4) | 11.3 (10.3-14.3) | 0.239 |
| NFL, ng/ml | 11.8 (7.5-16.4) | 21.4 (16.7-26.8) | **<**0.001 |
| T5 | n=69 | n=14 |  |
| S100β, pg/ml | 2876.0 (2329.0-3652.0) | 3125.5 (2567.0-4663.0) | 0.141 |
| NSE, ng/ml | 11.4 (9.6-12.8) | 12.3 (9.8-12.9) | 0.616 |
| NFL, ng/ml | 12.5 (9.2-16.6) | 18.0 (13.5-24.2) | **<**0.001 |
| T6 | n=62 | n=13 |  |
| S100β, pg/ml | 2832.5 (2323.5-3452.5) | 3448.0 (2574.0-4307.0) | 0.090 |
| NSE, ng/ml | 11.4 (10.0-12.8) | 11.9 (9.9-13.6) | 0.497 |
| NFL, ng/ml | 14.0 (8.3-17.3) | 10.5 (8.5-20.0) | 0.862 |
| T7 | n=50 | n=9 |  |
| S100β, pg/ml | 3000.5 (2356.2-3897.8) | 3794.0 (2925.0-4798.0) | 0.117 |
| NSE, ng/ml | 11.2 (9.7-13.2) | 12.2 (10.9-14.0) | 0.773 |
| NFL, ng/ml | 12.5 (7.9-16.8) | 10.0 (6.2-21.1) | 0.709 |

Data represent median(interquartile range).
